# Impact of benznidazole treatment on the functional response of *Trypanosoma cruzi* antigen-specific CD4^+^CD8^+^ T cells in chronic Chagas disease patients

**DOI:** 10.1371/journal.pntd.0006480

**Published:** 2018-05-11

**Authors:** Elena Pérez-Antón, Adriana Egui, M. Carmen Thomas, Concepción J. Puerta, John Mario González, Adriana Cuéllar, Manuel Segovia, Manuel Carlos López

**Affiliations:** 1 Instituto de Parasitología y Biomedicina López-Neyra, Consejo Superior de Investigaciones Científicas; Granada, Spain; 2 Laboratorio de Parasitología Molecular, Pontificia Universidad Javeriana; Bogotá, Colombia; 3 Grupo de Ciencias Básicas Médicas, Facultad de Medicina, Universidad de los Andes; Bogotá, Colombia; 4 Grupo de Inmunobiología y Biología Celular, Pontificia Universidad Javeriana; Bogotá, Colombia; 5 Unidad Regional de Medicina Tropical, Hospital Virgen de la Arrixaca; Murcia, Spain; Instituto de Ciências Biológicas, Universidade Federal de Minas Gerais, BRAZIL

## Abstract

**Background:**

Chagas disease is caused by *Trypanosoma cruzi*. The persistence of the parasite is associated with the disease chronicity and the impairment of the cellular immune response. It has been reported that the CD4^+^CD8^+^ T cell population expands in chronic Chagas disease patients. Few studies have focused on this subset of cells, and very little is known about the impact of antiparasitic treatment on this population.

**Methodology:**

Thirty-eight chronic Chagas disease patients (20 asymptomatic and 18 symptomatic) and twelve healthy controls were enrolled in this study. Peripheral blood mononuclear cells were stimulated with soluble *T*. *cruzi* antigens to analyze the production of cytokines and cytotoxic molecules by CD4^+^CD8^+^ T cells before and after benznidazole treatment. Additionally, expression and co-expression of five inhibitory receptors in these patients after treatment were studied using a multiparameter flow cytometry technique.

**Principal findings:**

The frequency of CD4^+^CD8^+^ T cells was higher in chronic Chagas disease patients compared with healthy donors. Furthermore, a higher ratio of CD4^+^CD8^low^/CD4^+^CD8^high^ subpopulations was observed in chronic Chagas disease patients than in healthy donors. Additionally, CD4^+^CD8^+^ T cells from these patients expressed and co-expressed higher levels of inhibitory receptors in direct proportion to the severity of the pathology. Benznidazole treatment reduced the frequency of CD4^+^CD8^+^ T cells and decreased the ratio of CD4^+^CD8^low^/CD4^+^CD8^high^ subpopulations. The co-expression level of the inhibitory receptor was reduced after treatment simultaneously with the enhancement of the multifunctional capacity of CD4^+^CD8^+^ T cells. After treatment, an increase in the frequency of *T*. *cruzi* antigen-specific CD4^+^CD8^+^ T cells expressing IL-2 and TNF-α was also observed.

**Conclusions:**

CD4^+^CD8^+^ T cells could play an important role in the control of *T*. *cruzi* infection since they were able to produce effector molecules for parasite control. Benznidazole treatment partially reversed the exhaustion process caused by *T*. *cruzi* infection in these cells with an improvement in the functional response of the *T*. *cruzi* antigen-specific CD4^+^CD8^+^ T cells.

## Author summary

Chagas disease is a neglected tropical disease caused by the intracellular parasite *Trypanosoma cruzi*. The persistence of the parasite leads to deterioration of the host immune response, which is known as an exhaustion process. This process affects T cell populations, leading to increased expression of inhibitory receptors that leads to a dysfunctional ability to respond to the parasite. CD4^+^CD8^+^ T cells form a poorly studied population of T cells in the context of Chagas disease. In this study, as in others previously reported, an increase in the frequency of these cells was observed in chronic Chagas disease patients. In addition, CD4^+^CD8^+^ T cells from chronic Chagas disease patients underwent stronger exhaustion processes with more severe disease pathology. A higher level of expression and co-expression of inhibitory receptors was observed in these cells in symptomatic compared with asymptomatic patients. Furthermore, we evaluated whether antiparasitic treatment affected the population of CD4^+^CD8^+^ T cells. Our results showed that after treatment, the functional capacity of these cells against the parasite improved. Concomitantly, a partial reversion of this exhaustion process occurred since the co-expression of inhibitory receptors decreased in CD4^+^CD8^+^ T cells from chronic patients after treatment.

## Introduction

Even 100 years after Chagas disease was reported for the first time by Carlos Chagas, the infection remains an important public health problem in Latin America, with a high social and economic burden [1] and with a growing global impact [2]. The World health organization (WHO) estimates that approximately 8–10 million people worldwide are infected with *Trypanosoma cruzi*, the causal agent of Chagas disease, which causes approximately 20,000 deaths per year and is the leading cause of infectious myocarditis [3]. The main trypanocidal drug used to treat Chagas disease is benznidazole (BNZ). Side effects associated with BNZ therapy occurred in 30% to 87% of patients under the form of cutaneous, gastrointestinal or neurological disorders [4–6]. The effectiveness of BNZ during the chronic phase of the disease remains controversial. However, several studies have provided evidence supporting the benefit of BNZ during the chronic phase of Chagas disease based on its ability to improve the immune system capacity against the parasite [7, 8] and prevent the development of cardiomyopathy [9–11]. Moreover, the WHO as well as some systematic reviews recommend the use of antiparasitic treatment during the chronic phase of disease [12–16]. Taking all of this information into account, a key aspect to be considered in the fight against this disease is the need to identify biomarkers of therapeutic efficacy and their use as a new tool to facilitate the follow-up of treated patients [17].

*T*. *cruzi* infection usually initiates with high parasitemia in blood that leads a strong immune response to partially control the infection, although it rarely resolves it completely. The parasite manages to hide in tissues that are less accessible to the immune response, resulting in infection chronicity [18]. Most patients maintain an asymptomatic chronic disease over years or even decades, but approximately 30–40% develop a symptomatic chronic phase [19]. In chronic infectious diseases, T cells undergo an important process known as cellular exhaustion [20]. The exhaustion process is produced by a continuous exposure to pathogen antigens that leads to a dysfunctional response of the T cells via an impaired ability to produce cytokines and cytotoxic molecules against the infectious agent, accompanied by a progressive increase in the expression and co-expression of inhibitory receptors on the membrane of antigen-specific T cells [20, 21]. The exhaustion process in Chagas disease occurs in CD8^+^ and CD4^+^ T cells [22, 23] and has been described be more dramatic during more severe stages of disease [23]. Recently, anti-*T*. *cruzi* treatment has been shown to reduce this process of exhaustion in CD8^+^ T cells in chronic Chagas disease patients [24].

Circulating T cells are considered the key components of the adaptive immune system, and principally CD8^+^ and CD4^+^ T cells are the best described and known populations functioning in the control of *T*. *cruzi* infection [25–27]. Other T cells components require further studies to achieve a better understanding of their functions in the immune system, including CD4^+^CD8^+^ peripheral T cells, which were described by Blue et al. as a population representing approximately 3% of lymphocytes in human blood [28]. Subsequent studies have characterized this cellular population in detail, but more information is still needed. Several groups have shown that CD4^+^CD8^+^ T cells comprise mature T cells that are capable of being activated [29–32] and able to respond to specific antigen-producing cytokines and cytotoxic molecules and to migrate to inflamed tissues [30–32]. Thus, their role has been studied in viral chronic infectious diseases such as HIV [29] and HCV [31]. In the context of Chagas disease, Giraldo et al. recently described the population of CD4^+^CD8^+^ T cells in patients with chronic *T*. *cruzi* infection. They observed an increased frequency of this circulating subset of cells in chronic chagasic patients and demonstrated that CD4^+^CD8^+^ T cells responded to *T*. *cruzi* antigens and expressed greater amounts of activation markers and perforin than healthy subjects. Furthermore, these cells were found in the inflammatory infiltrate of cardiac tissue from a patient who underwent cardiac transplant [33], suggesting they could play an important role in the control of Chagas disease.

In the present study, we studied the population of CD4^+^CD8^+^ T cells in the context of chronic Chagas disease. Their ability to respond to *T*. *cruzi* antigens and the mechanism underlying the exhaustion process in these T cells were analyzed. Furthermore, the impact of benznidazole treatment on this CD4^+^CD8^+^ T cell population and whether improved the antigen-specific response and reversed the exhaustion process in these CD4^+^CD8^+^ T cells were assessed. This type of study can lead to the identification of new biomarkers that are useful and necessary for evaluating the therapeutic efficacy of known and new drugs. Furthermore, it can improve knowledge concerning all components of the immune system that play a key role in the control of *T*. *cruzi* infection.

## Methods

### Study population

This study enrolled 38 adult patients diagnosed with chronic Chagas disease (cChD). These patients were clinically characterized based on the Kuschnir classification of Chagas disease [34] and included 20 asymptomatic patients (IND) and 18 patients with heart damage (CCC) (G1, n = 8; G2, n = 6 and G3, n = 4). The patients were residents of Spain from endemic areas who were diagnosed with Chagas disease following WHO criteria based on two conventional serological tests (Chagas ELISA, Ortho Clinical Diagnosis and Inmunofluor Chagas, Biocientífica, Argentina) at the Hospital Virgen de la Arrixaca (Murcia-Spain) and who had never received any treatment for their disease. Patients with evidence of gastrointestinal disorders were excluded. All patients were treated with benznidazole 5 mg/kg/d for 60 days [35], and clinical follow-up was performed during the entire 48-month study period. Twelve healthy donors (HD) were also included. During this study the patients did not show any clinical changes that could be associated with progression of Chagas disease.

### Ethical considerations

The Ethic Committees from the Hospital Virgen de la Arrixaca, Murcia-Spain, and the Consejo Superior de Investigaciones Científicas (CSIC), Spain, approved the protocols used in this study. Signed informed consent was obtained from all individuals prior to their inclusion in the study.

### Blood samples

Peripheral blood samples of 30 mL from each individual were collected by venipuncture into EDTA-containing tubes. Samples were collected prior to treatment administration (T0) and at two post-treatment times: after 9–12 months (T1) and after 24–48 months (T2). Peripheral blood mononuclear cells (PBMC) were purified as previously described [36], stored in inactivated fetal bovine serum (iFBS) (Gibco, Grand Island, NY) with 10% DMSO and cryopreserved in liquid nitrogen until used.

### DNA purification and PCR amplification

Genomic DNA purification from the peripheral blood samples and PCR amplification for parasite detection were performed as previously described by Murcia et al. [37].

### Isolation of *T*. *cruzi* soluble antigens

To obtain *T*. *cruzi* soluble antigens (*Tc*SA), Rhesus Monkey Kidney Epithelial Cells (LLC-MK2 line; CCL-7, Manassas, VA) were cultured in RPMI-1640 supplemented with 2 mM L-glutamine (Gibco), 10% iFBS and 50 μg/mL gentamicin (Thermo Fisher Scientific, Waltham, Massachusetts, USA) at 37°C in a humidified atmosphere with 5% CO_2_. The cells were grown until they formed a monolayer, which was infected with *T*. *cruzi* trypomastigote forms (MHOM/BR/1950/ Y isolated strain) obtained from *T*. *cruzi* experimentally infected mice (parasite:cell ratio of 4:1). After 96 h, the parasites were recovered in trypomastigote and amastigote forms from infected-culture supernatants by centrifugation and subsequently washed with 1x PBS at pH = 7.2. Parasites were suspended at 1x10^6^ parasites/μl in lysis buffer (50 mM Tris-HCl at pH 7.4, 50 mM NaCl, 0.005% NP-40, 1 mM PMSF, and 1 μg/mL leupeptin) and sonicated 3 times with pulses of 50–62 KHz for 40-s time intervals of 20 s. Soluble protein extracts were obtained by centrifugation at 10,000 rpm for 20 min at 4°C. The protein concentration was determined using the micro BCA protein assay kit (Thermo Fisher Scientific), and the protein profile was analyzed by SDS-PAGE followed by Coomassie blue staining (Gibco).

### Antibodies

The following conjugated Abs were used for cell surface staining: CD3-Pacific Blue (clone UCHT1), CD4-Alexa Fluor 700 (clone RPA-T4), CD8-APC-H7 (clone SK1), CD160-Alexa Fluor 647 (clone BY55), T-cell immunoglobulin and mucin-domain containing-3 (TIM-3)-PE-CF594 (clone 7D3), 2B4-FITC (clone 2–69) (BD Biosciences, San Jose, CA), and programmed cell death protein-1 (PD-1)-PE (clone J105) (Thermo Fisher Scientific). Conjugated Abs for intracellular staining included the following: cytotoxic T lymphocyte antigen-4 (CTLA-4)-PE-Cy5 (clone BNI3), granzyme B-PE-CF594 (clone GB11), interferon-gamma (IFN-γ)-PE-Cy7 (clone B27), interleukin-2 (IL-2)-APC (clone MQ1-17H12), tumor necrosis factor-alpha (TNF-α)-Alexa Fluor 488 (clone MAb11) (BD Biosciences, San Diego, CA), and perforin-PE (clone B-D48) (Abcam, Cambridge, UK). All conjugated Abs were titrated and multicolor panels for flow cytometry assays were approached as previously reported [38]. The following purified (No azide/Low endotoxin) Abs were used in cultured cells: CD28 (clone CD28.2) and CD49d (clone 9F10) (BD Biosciences).

### Intracellular cytokine and cytotoxic molecules detection by flow cytometry assays in PBMC after stimulation with *T*. *cruzi* soluble antigens

PBMCs were cultured in RPMI-1640 supplemented with 2 mM L-glutamine, 10% iFBS and 50 μg/mL gentamicin. PBMCs were cultured at 1x10^6^ cells/mL in the presence of anti-CD28 (1 μg/mL) and anti-CD49d (1 μg/mL) with *Tc*SA (1 μg/mL) or without *Tc*SA (considered the basal response). The cells were incubated for 10 h at 37°C in a humidified atmosphere with 5% CO_2_, with the last 9 h in the presence of brefeldin A (1 μg/mL) and monensin (2 μM) (BD Bioscience). To evaluate intracellular cytokine production, at least 1x10^6^ cells were stained for each condition. First, PBMCs were stained with a viability marker, LIVE/DEAD Fixable Aqua (Invitrogen, Eugene, OR), for 20 min in darkness at RT. The cells were stained with anti-CD3, anti-CD4, and anti-CD8 Abs. After washing, the cells were fixed and permeabilized with Cytofix/Cytoperm (BD Biosciences). Intracellular staining was performed with anti-granzyme B, anti-IFN-γ, anti-IL-2, anti-TNF-α, and anti-perforin Abs for 30 min at 4°C. Finally, 100,000 lymphocytes were acquired according to FCS/SSC parameters through a FACSAria III flow cytometer (BD Biosciences) for each condition. The data file was subsequently analyzed using FlowJo 9.3.2 software (Tree Star, Ashland, OR) to determine the percentages and mean fluorescence intensity (MFI) and to assess co-expression populations using a Boolean gating strategy. The gating strategy used is presented in S1 Fig. The positivity for each marker was determined using the fluorescence minus one (FMO) and isotype control stains. To obtain a robust data analysis, patients who at the time of pretreatment had a frequency of CD4^+^CD8^+^ T cells greater than 0.3% and greater than 100 acquisition events for that population were selected for the study.

### Identification of inhibitory receptor expression by flow cytometry

PBMCs were cultured in the same medium and under the same conditions as previously described for the cytokine detection assay. The cells were cultured in the presence of anti-CD28 and anti-CD49d and with *Tc*SA (1 μg/mL) for 10 h. The cells were stained with the following cell surface Abs: anti-CD3, anti-CD4, and anti-CD8, anti-2B4, anti-CD160, anti-PD-1, and anti-TIM-3. After cell permeabilization, intracellular staining was performed with anti-CTLA-4 Ab. The cells were acquired and analyzed as described in the previous assay.

### Statistical analysis

Statistical analyses were performed using GraphPad Prism version 6.0 software (GraphPad Software, San Diego, CA). Nonparametric tests were used to test for statistical significance. The Mann Whitney U test was used to carry out comparisons among patients and healthy donors or CCC and IND groups. The Wilcoxon test was used to study the post-treatment evolution. An additional analysis was performed to compare co-expression pie charts using 10,000 permutations calculated with SPICE version 5.3 software (the National Institutes of Health, Bethesda, MD). Statistical significance was assigned to values of ρ<0.05, and the symbology used was ρ<0.05 (*), ρ<0.1 (**), ρ<0.001 (***) and ρ<0.0001 (****). The box-plot graphics were performed by GraphPad Prism version 6.0 software and represent all values (minimum to maximum). The boxes represent the 25^th^ to 75^th^ percentiles.

## Results

### Circulating population of CD4^+^CD8^+^ T cells in chronic Chagas disease patients and effect of benznidazole treatment on this T cell subset

**Fig 1:**
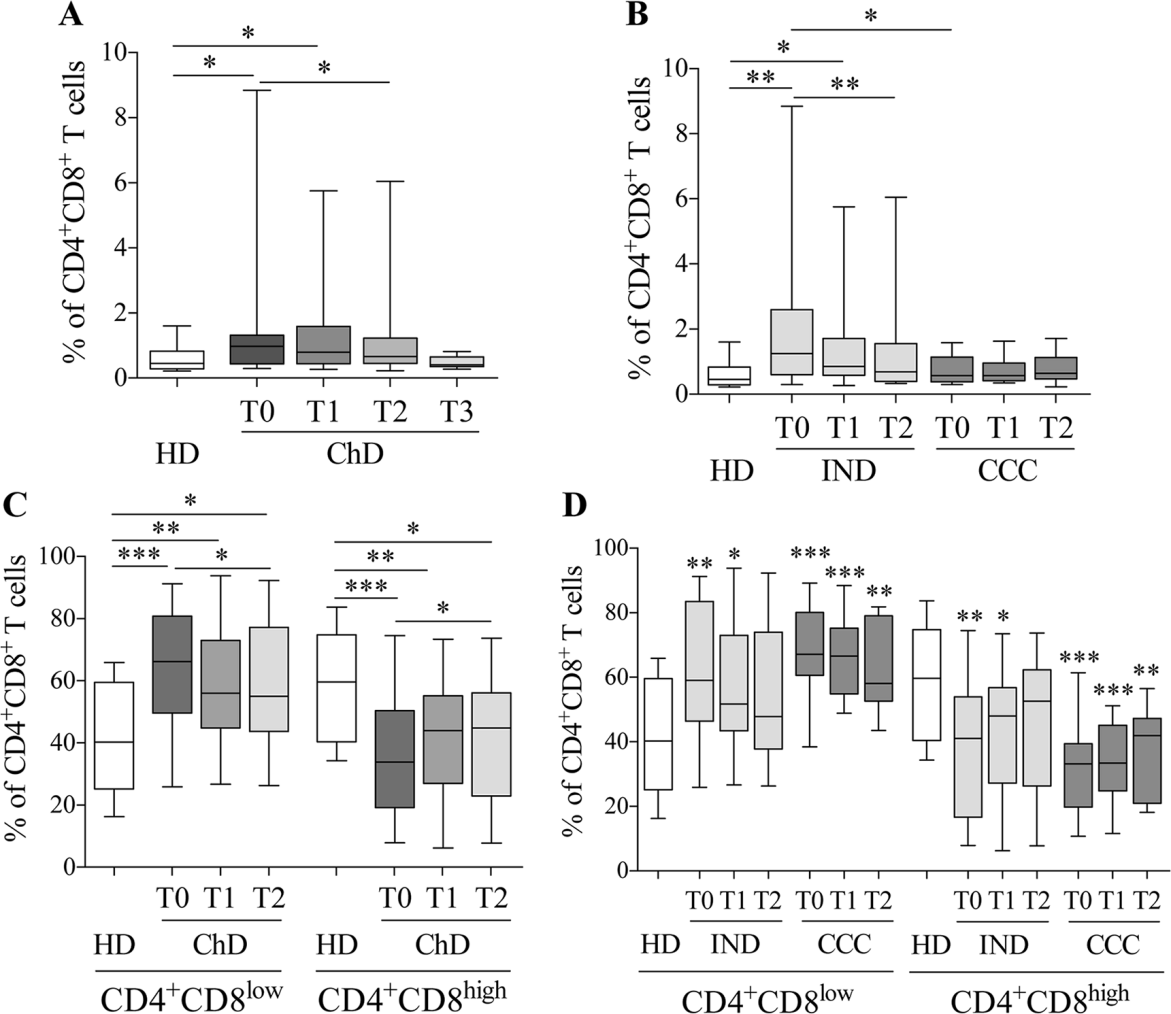
Percentage of circulating CD4^+^CD8^+^ T cells, CD4^+^CD8^low^ and CD4^+^CD8^high^ subpopulations pre and post treatment. Frequencies were evaluated at pre-treatment (T0) and at two times points after treatment: 9–12 months (T1) and 24–48 months (T2) in cChD patients (IND and CCC) and HD. **(A)** Follow-up of the CD4^+^CD8^+^ T cell frequency in HD and cChD after benznidazole treatment. **(B)** Frequency of CD4^+^CD8^+^ T cells in HD, IND and CCC before and after treatment. **(C)** Frequencies of CD4^+^CD8^low^ and CD4^+^CD8^high^ T cells were evaluated at the times points indicated in cChD and HD. **(D)** The percentages of the subpopulations of CD4^+^CD8^+^ T cells (CD4^+^CD8^low^ and CD4^+^CD8^high^) were assessed before and after treatment in IND, CCC and HD. The statistical symbols (*) above the whiskers correspond to differences between HD and cChD at the different time points assessed. Statistical analyses were carried out using the Mann-Whitney U test and Wilcoxon test. Statistically significant differences are indicated by (*) ρ<0.05, (**) ρ<0.01 and (***) ρ<0.001. The study population was stratified by cChD (IND (n = 20), CCC (n = 18)) and HD (n = 12).

To study the CD4^+^CD8^+^ T cell subset, the frequency of CD4^+^CD8^+^ T cells in the CD3^+^ lymphocyte population was first determined in PBMC samples from 12 healthy donors (HD) and 36 (19 IND and 16 CCC) chronic Chagas disease patients (cChD) who had not received any anti-*T*. *cruzi* treatment (T0) and at 9–12 (T1) and 24–48 (T2) months post-treatment. A statistically higher frequency of CD4^+^CD8^+^ T cells was found in cChD than in HD (ρ = 0.014) (Fig 1A). When the analysis was performed according the severity of the disease, a higher frequency of CD4^+^CD8^+^ T cell was found in IND patients than in HD (ρ = 0.003) and CCC patients (ρ = 0.016) (Fig 1B).

As observed in Fig 1A and 1B, BNZ treatment had an impact on the percentage of the CD4^+^CD8^+^ T cell population. Thus, the percentages of CD4^+^CD8^+^ T cells in 38 cChD (20 IND and 18 CCC) at 9–12 (T1) and 24–48 (T2) months post-treatment showed (Fig 1A and 1B) a continuous decrease in the frequency of CD4^+^CD8^+^ T cells after treatment, as evidenced by an average 1.45% of cells in untreated patients (T0) and 1.17 and 1.07% in T1 and T2, respectively. This decrease was statistically significant between T0 and T2 (ρ = 0.043) (Fig 1A). In addition, a statistically significant decrease in the frequency of CD4^+^CD8^+^ T cells from IND (ρ = 0.008, between T0 and T2), but not from CCC was observed after treatment. Subsequently, the subpopulations of CD4^+^CD8^+^ T cells were studied in these subjects according to low or high CD8 expression (Fig 1C and 1D). A higher percentage of CD4^+^CD8^low^ T cells and lower percentage of CD4^+^CD8^high^ T cells were detected in cChD patients (T0) compared with HD (ρ = 0.0003 and ρ = 0.0002, respectively) (Fig 1C). Furthermore, the differences in the frequency of both CD4^+^CD8^low^ and CD4^+^CD8^high^ subsets were higher between CCC and HD (ρ = 0.0002) than between IND and HD (ρ = 0.007) (Fig 1D). Interestingly, after BZN administration it was observed a decrease in the frequency of CD4^+^CD8^low^ T cells (ρ = 0.013, T2 *versus* T0) and an increase in the frequency of CD4^+^CD8^high^ T cells (ρ = 0.014, T2 *versus* T0) in both IND and CCC (Fig 1C and 1D). Therefore, after treatment, the ratio of the frequency of CD4^+^CD8^high^/CD4^+^CD8^low^ subpopulations from IND and CCC chronic patients tended to be similar to that detected in HD (Fig 1D).

### Expression and co-expression of inhibitory receptors in the circulating population of CD4^+^CD8^+^ T cells in *Trypanosoma cruzi* chronic infection patients

**Fig 2:**
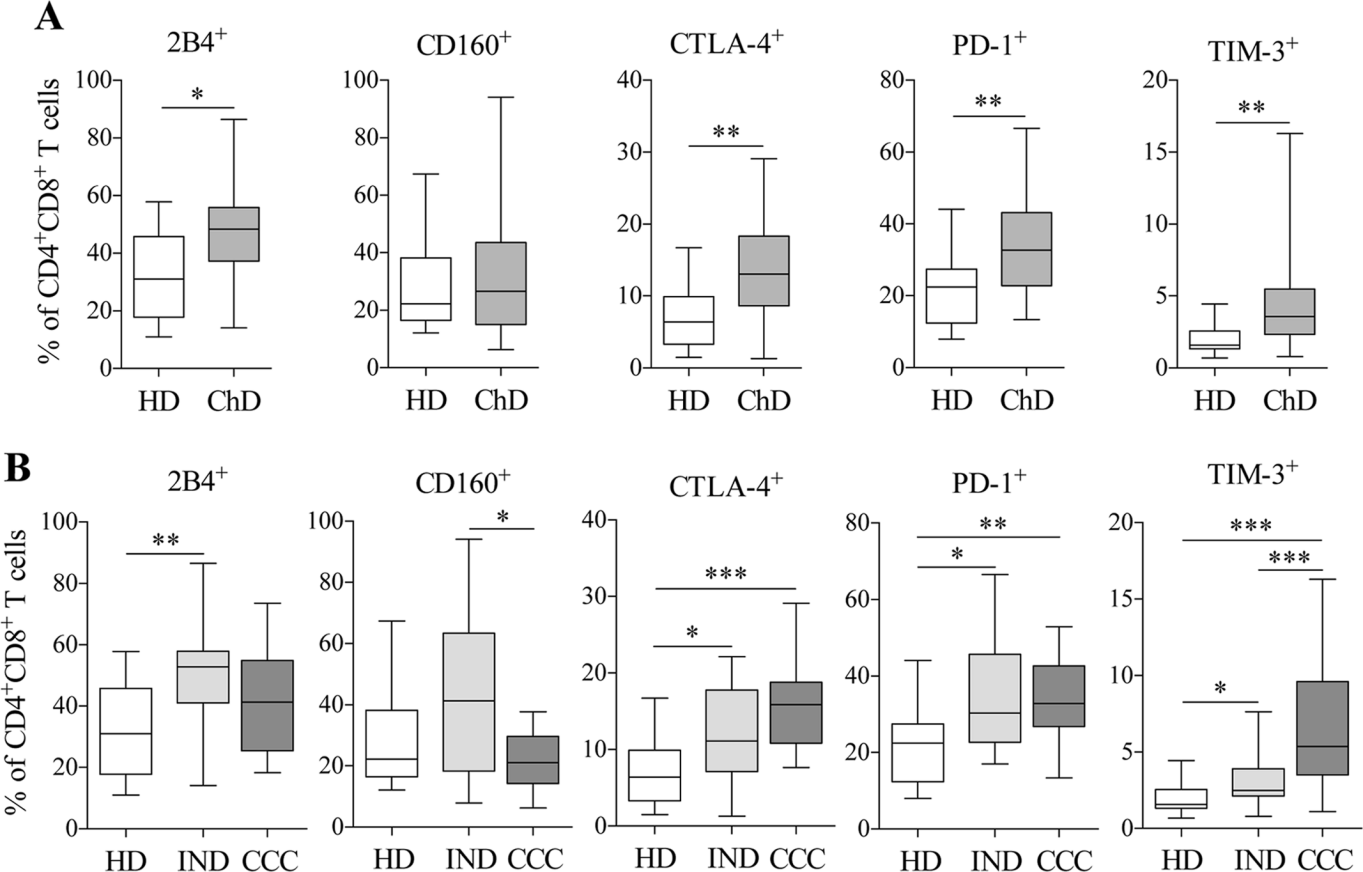
Percentage of CD4^+^CD8^+^ T cells expressing 2B4, CD160, CTLA-4, PD-1 and TIM-3. The study was carried out in cChD patients (IND and CCC) and HD. **(A)** Individual expression levels of inhibitory receptors in HD (n = 12) and cChD (n = 35). **(B)** Frequency of CD4^+^CD8^+^ T cells expressing inhibitory receptors in HD (n = 12), IND (n = 19) and CCC (n = 16). Statistical analyses were carried out using the Mann-Whitney U test. Statistically significant differences are indicated by (**) ρ<0.01 and (***) ρ<0.001.

PBMCs isolated from 35 cChD (19 IND and 16 CCC) and 12 HD were stained with conjugated Abs as described in “Materials and Methods” to assess the expression and co-expression of inhibitory receptor in CD4^+^CD8^+^ T cells. The frequency of CD4^+^CD8^+^ T cells expressing 2B4, CD160, CTLA-4, PD-1 and TIM-3 was determined in cChD and HD (Fig 2A). As shown in Fig 2A, the frequency of CD4^+^CD8^+^ T cells expressing 2B4^+^ (ρ = 0.011), CTLA-4^+^ (ρ = 0.002), PD-1^+^ (ρ = 0.005) and TIM-3^+^ (ρ = 0.002) was higher in cChD than in HD. When this population was separated in IND and CCC (Fig 2B), CCC showed a higher frequency of cells expressing CTLA-4 (ρ = 0.0006), PD-1 (ρ = 0.008) and TIM-3 (ρ = 0.001) compared with HD, as well as cells expressing TIM-3 compared with IND (ρ = 0.0005). The IND patients showed a higher frequency of CD4^+^CD8^+^ T cells expressing 2B4 (ρ = 0.004), CTLA-A (ρ = 0.031), PD-1 (ρ = 0.020) and TIM-3 (ρ = 0.047) than HD. In contrast, a statistically lower frequency of CD4^+^CD8^+^ T cells expressing CD160 in CCC than in IND (ρ = 0.022) was found, although it did not extend to HD. Furthermore, an analysis was performed with the data corresponding to the MFI of each marker under study between cChD (IND and CCC) and HD (S2 Fig). The results showed that CD4^+^CD8^+^ T cells in cChD expressed higher levels of 2B4 (ρ<0.0001), CTLA-4 (ρ = 0.014), PD-1 (ρ = 0.0007) and TIM-3 (ρ<0.05) compared with HD. A separate analysis of IND and CCC (S2B Fig) revealed a higher expression level of the inhibitory receptors 2B4 (ρ<0.0001), CTLA-4 (ρ = 0.028), PD-1 (ρ = 0.0002) and TIM-3 (ρ = 0.0004) in CCC compared with HD and a higher expression level of TIM-3 compared with IND (ρ = 0.002). The expression level of CD160 in CD4^+^CD8^+^ T cells was higher in IND compared with CCC (ρ = 0.022) and HD (ρ = 0.021). Furthermore, expression of the CD160 marker in the subpopulations of CD4^+^CD8^+^ T cells (CD4^+^CD8^high^ and CD4^+^CD8^low^) in IND and CCC was examined (S3 Fig). A more significant difference was observed in the frequency of cells expressing CD160 within the subpopulation of CD4^+^CD8^high^ T cells between IND and CCC (ρ<0.0001).

**Fig 3:**
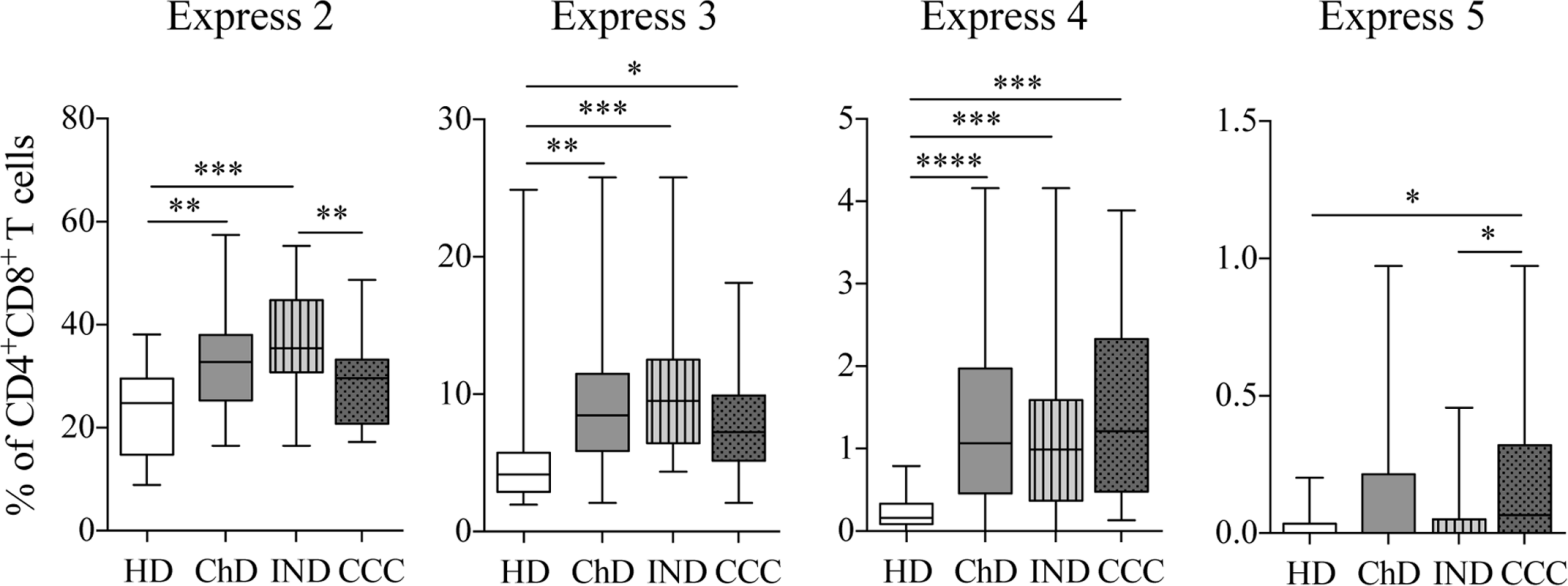
Inhibitory receptor co-expression on CD4^+^CD8^+^ T cells in cChD, IND, CCC, and HD. Inhibitory receptors assessed were 2B4, CD160, CTLA-4, PD-1 and TIM-3. Statistical analyses were carried out using the Mann-Whitney U test. Statistically significant differences are indicated by (*) ρ<0.05, (**) ρ<0.01, (***) ρ<0.001 and (****) ρ<0.0001. The study population was stratified by cChD (IND (n = 19) and CCC (n = 15)) and HD (n = 12).

Likewise, the co-expression of inhibitory receptors (2B4, CD160, CTLA-4, PD-1 and TIM-3) in CD4^+^CD8^+^ T cells from cChD and HD was examined. As shown in Fig 3, there was a higher percentage of CD4^+^CD8^+^ T cells co-expressing 2 (ρ = 0.003), 3 (ρ = 0.001) and 4 (ρ<0.0001) inhibitory molecules in cChD *versus* HD. When the cells were analyzed separately in IND and CCC (Fig 3), CCC presented a higher frequency of CD4^+^CD8^+^ T cells co-expressing 5 inhibitory receptors and a lower frequency of cells co-expressing 2 inhibitory receptors compared with IND (ρ = 0.032 and ρ = 0.007, respectively). Altogether, these results revealed higher expression and co-expression of inhibitory receptors in the CD4^+^CD8^+^ T cell subset from chronic Chagas disease patients than from healthy donors.

### The functional and multifunctional capacity of circulating CD4^+^CD8^+^ T cells in chronic Chagas disease patients in response to *Trypansoma cruzi* antigens

**Fig 4:**
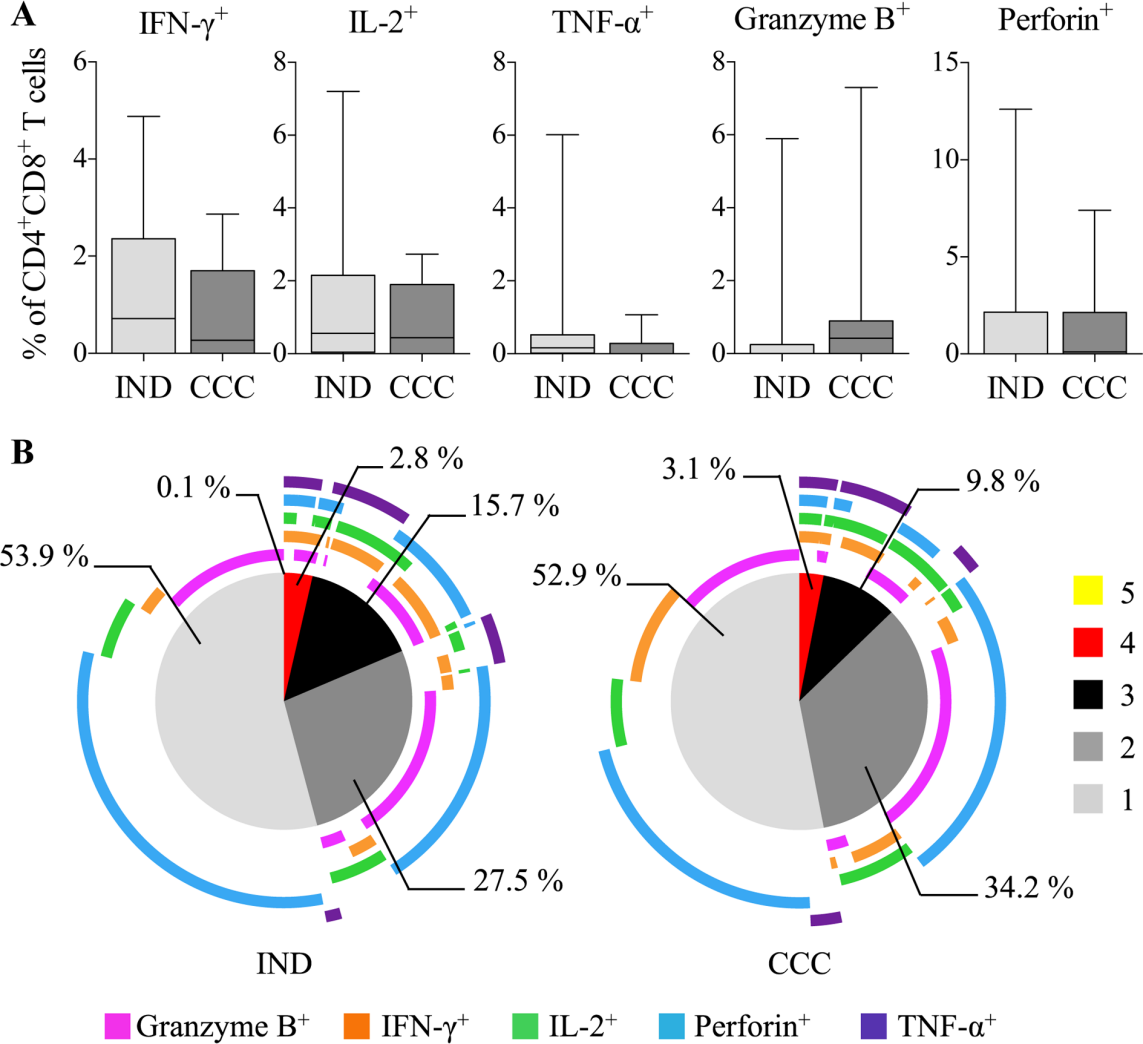
Functional and multifunctional capacity of *T*. *cruzi*-specific CD4^+^CD8^+^ T cells in IND and CCC. **(A)** Frequency of CD4^+^CD8^+^ T cells producing IFN-γ, IL-2, TNF-α, perforin or granzyme B against *Tc*SA, in IND and CCC. **(B)** Multifunctional activity profiles of *T*. *cruzi*-specific CD4^+^CD8^+^ T cells in IND and CCC. The color of the portions of the pie charts depicts the number of molecules produced by CD4^+^CD8^+^ T cells in response to *T*. *cruzi* antigens. The arcs of the pie charts represent the proportion of cells expressing each one of the molecules under study. The cChD were divided into IND (n = 13) and CCC (n = 15).

The *T*. *cruzi* antigen-specific functional activity of CD4^+^CD8^+^ T cells from 13 IND and 15 CCC was assessed as described in the “Materials and Methods” section. The percentages of CD4^+^CD8^+^ T cells producing intracellular cytokines (IFN-γ, IL-2 and TNF-α) and cytotoxic molecules (perforin and granzyme B) in response to *Tc*SA were thus determined. The percentage of CD4^+^CD8^+^ T cells expressing these molecules without stimulation (basal response) was subtracted from that obtained following antigen stimulation (Fig 4A). A slightly higher, but not significant, frequency of CD4^+^CD8^+^ T cells was found expressing IFN-γ and/or TNF-α in IND compared with CCC, and a higher frequency of CD4^+^CD8^+^ T cells expressing granzyme B in CCC compared with IND. Furthermore, the multifunctional profile was evaluated in CD4^+^CD8^+^ T cells in response to *Tc*SA based on the production of the following molecules in both IND and CCC: IFN-γ, IL-2, TNF-α, perforin and granzyme B. As shown in Fig 4B, a higher frequency of CD4^+^CD8^+^ T cells producing 3 effector molecules was observed in IND compared with CCC (15.7% *versus* 9.8%). Remarkably, the 41.2% of these cells from IND were expressing IFN-γ, perforin and granzyme B. However, only a 1.1% of these cells from CCC expressed IFN-γ, perforin and granzyme B (Fig 4B).

### Effect of BNZ treatment on the expression and co-expression of inhibitory receptors by CD4^+^CD8^+^ T cells in Chagas disease patients

**Fig 5:**
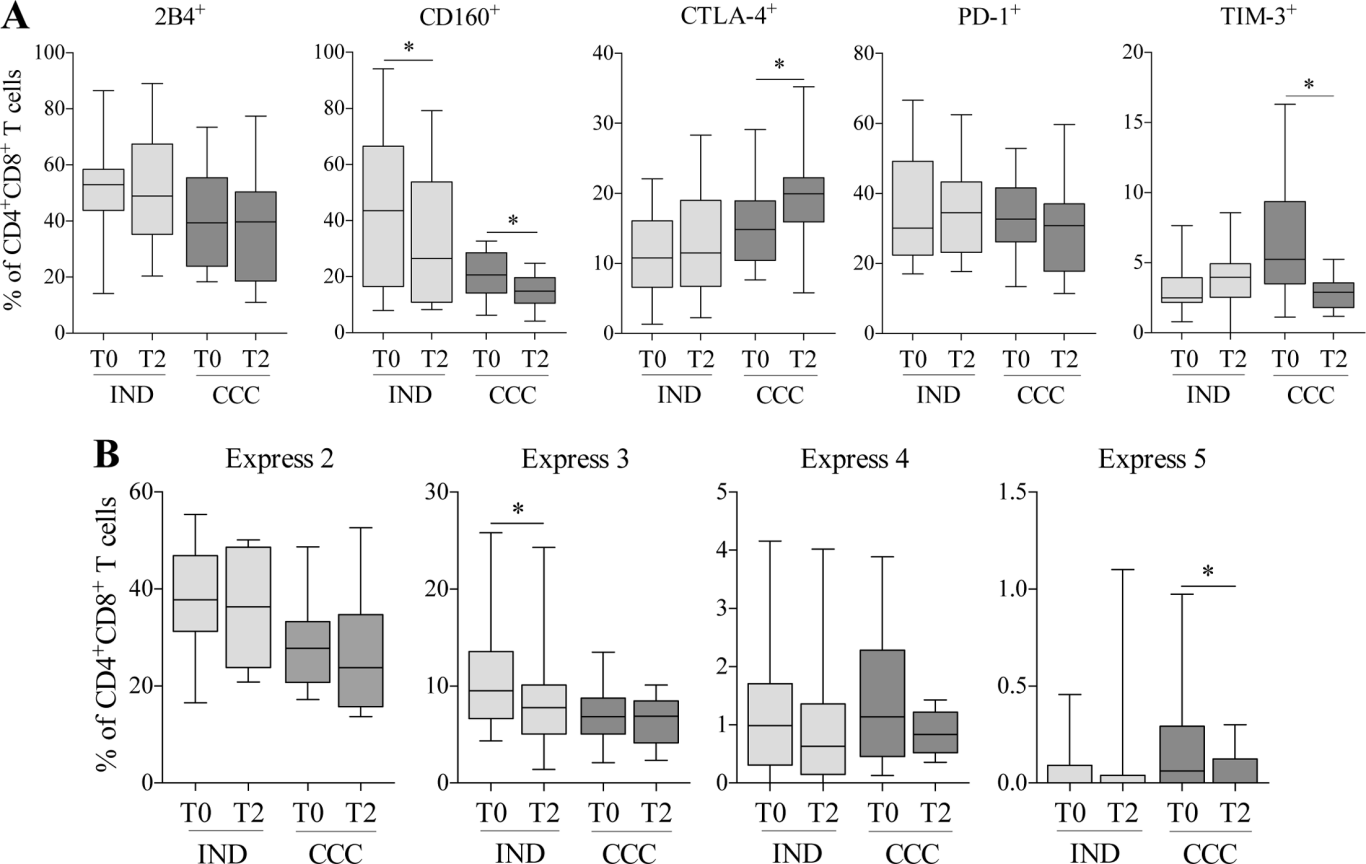
Effect of benznidazole on the expression and co-expression of inhibitory receptors in IND and CCC. 2B4, CD160, CTLA-4, PD-1 and TIM-3 were the inhibitory receptors assessed. Frequencies were evaluated at pre-treatment (T0) and 24–48 months after treatment (T2) in cChD patients (IND and CCC). **(A)** Individual expression of inhibitory receptors in IND and CCC before and after treatment. **(B)** Frequency of CD4^+^CD8^+^ T cells expressing 2, 3, 4 or 5 inhibitory receptors in IND and CCC before and after treatment. Statistical analyses were carried out using the Wilcoxon test. Statistically significant differences are indicated by (*) ρ<0.05, (**) ρ<0.01 and (***) ρ<0.001. The cChD was grouped according T0, IND (n = 17) and CCC (n = 14); and T2, IND (n = 15) and CCC (n = 12).

The effect of BNZ on the expression and co-expression of inhibitory receptors by CD4^+^CD8^+^ T cells was evaluated in 17 IND and 17 CCC. A statistically significant decrease in the percentage of CD4^+^CD8^+^ T cells expressing CD160 in IND (ρ = 0.025) and CCC (ρ = 0.049) and TIM-3 in CCC (ρ = 0.049) was observed after treatment (Fig 5A). Interestingly, there was an increase in CTLA-4^+^ in CCC after treatment (ρ = 0.014). Furthermore, after treatment, there was a decrease in the frequency of cells co-expressing 2, 3, 4 and 5 markers in both IND and CCC (Fig 5B). The differences in the expression of the 3 molecules in IND and 5 molecules in CCC comparing the pre-treatment time (T0) and post-treatment (T2) were statistically significant (ρ = 0.022 and ρ = 0.031, respectively). Taken together, these results indicated that BNZ treatment largely decreased the percentage of CD4^+^CD8^+^ T cells expressing and co-expressing inhibitory receptor in IND and CCC subjects.

### Evolution of the functional capacity of CD4^+^CD8^+^ T cells from Chagas disease patients after BNZ treatment

**Fig 6:**
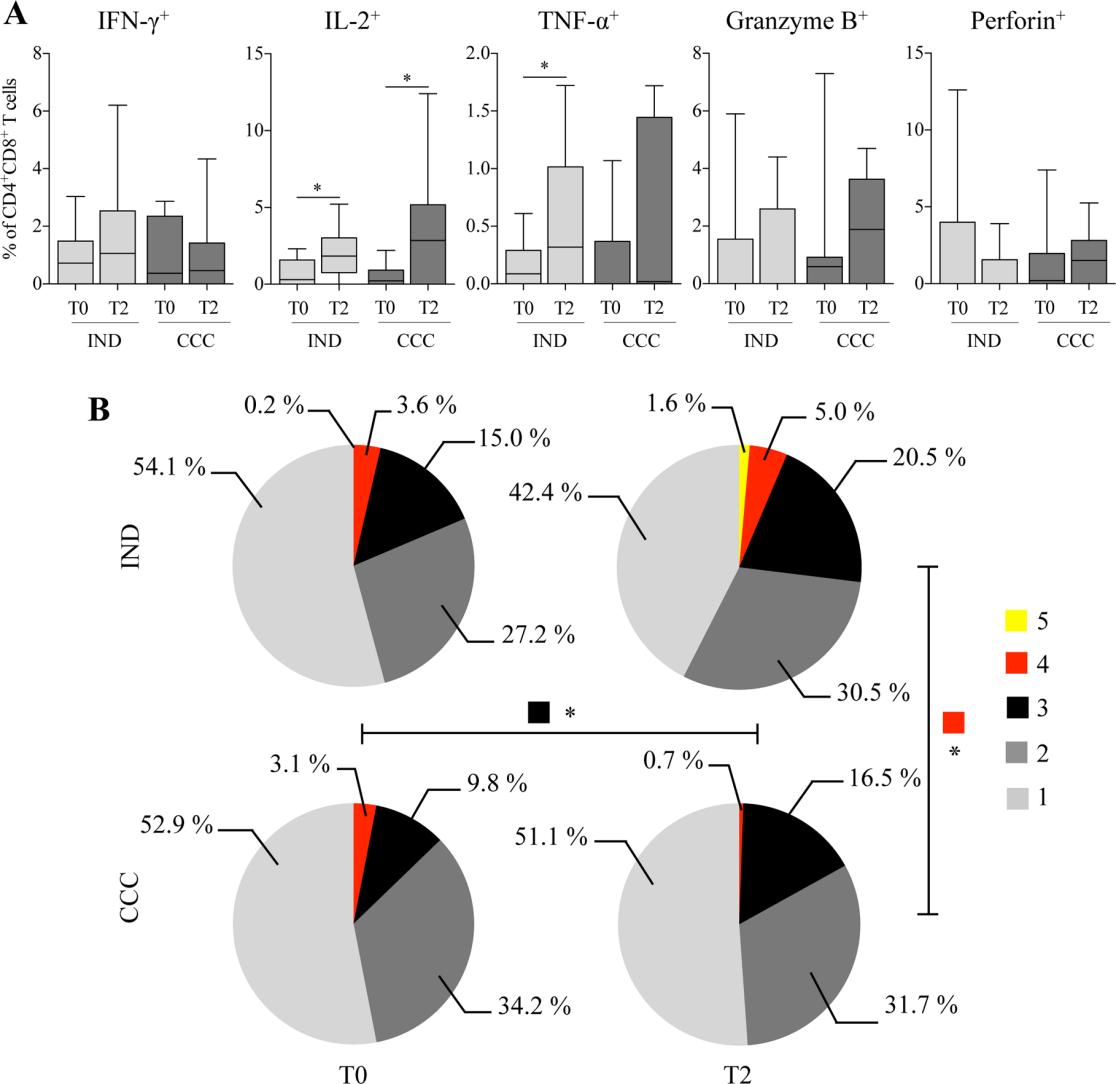
Impact of benznidazole on the functional and multifunctional capacity of *T*. *cruzi*-specific CD4^+^CD8^+^ T cells. The longitudinal study was carried out in IND and CCC patinets.**(A)** Frequency of CD4^+^CD8^+^ T cells producing IFN-γ, IL-2, TNF-α, perforin or granzyme B against *Tc*SA in IND and CCC before (T0) and 24–48 months after treatment. **(B)** Impact of the treatment on the multifunctional activity profiles of *T*. *cruzi*-specific CD4^+^CD8^+^ T cells in IND and CCC. Results obtained before (T0) and after treatment (T2). The color in the pie charts depicts the number of molecules produced by CD4^+^CD8^+^ T cells in response to *T*. *cruzi* antigens. Statistical analyses were carried out using the Wilcoxon and permutation test. Statistically significant differences are indicated by (*) ρ<0.05. The cChD was grouped into IND (n = 9) and CCC (n = 10).

To determine whether BZN treatment affected the functional capacity of CD4^+^CD8^+^ T cells from cChD, a longitudinal study was conducted in 9 IND and 10 CCC patients before and after treatment. The production of cytokines and cytotoxic molecules by CD4^+^CD8^+^ T cells after *Tc*SA stimulation was analyzed following subtraction of the basal response obtained without stimulation. Production of IFN-γ, TNF-α, IL-2, perforin and granzyme B by *T*. *cruzi* antigen-specific CD4^+^CD8^+^ T cells in IND and CCC was analyzed before (T0) and after treatment (T2) (Fig 6A). As shown in Fig 6A, there was a significant increase in the percentage of cells that produced IL-2 in IND (ρ = 0.023) and CCC (ρ = 0.039) and TNF-α in IND (ρ = 0.039). Likewise, after treatment, there was a slight increase in the cells that produced granzyme B in CCC. A slight increase in the frequency of CD4^+^CD8^+^IFN-γ^+^ T cells was also observed in IND, and in the percentage of cells that produce perforin in CCC. Furthermore, the multifunctionality of the *T*. *cruzi* antigen-specific CD4^+^CD8^+^ T cells was analyzed before and after treatment in IND and CCC (Fig 6B). *T*. *cruzi* antigen-specific CD4^+^CD8^+^ T cells from IND had a better multifunctional profile after treatment since there was a greater percentage of cells that produced 2 (27.2% to 30.5%), 3 (15% to 20.5%), 4 (3.6% to 5%) and 5 (0.2% to 1.6%) molecules in response to *Tc*SA (Fig 6B). CCC showed a significant increase in the proportion of *T*. *cruzi* antigen-specific CD4^+^CD8^+^ T cells that produced 3 molecules (ρ = 0.013; 9.8% to 16.5%) (Fig 6B). However, the percentage of CD4^+^CD8^+^ T cells that produced 4 molecules in response to *Tc*SA decreased in CCC after treatment (3.1% to 0.7%). Moreover, after treatment, the percentage of cells that produced 4 molecules was significantly higher in IND than in CCC (ρ = 0.018, 5% and 0.7%, respectively).

**Fig 7:**
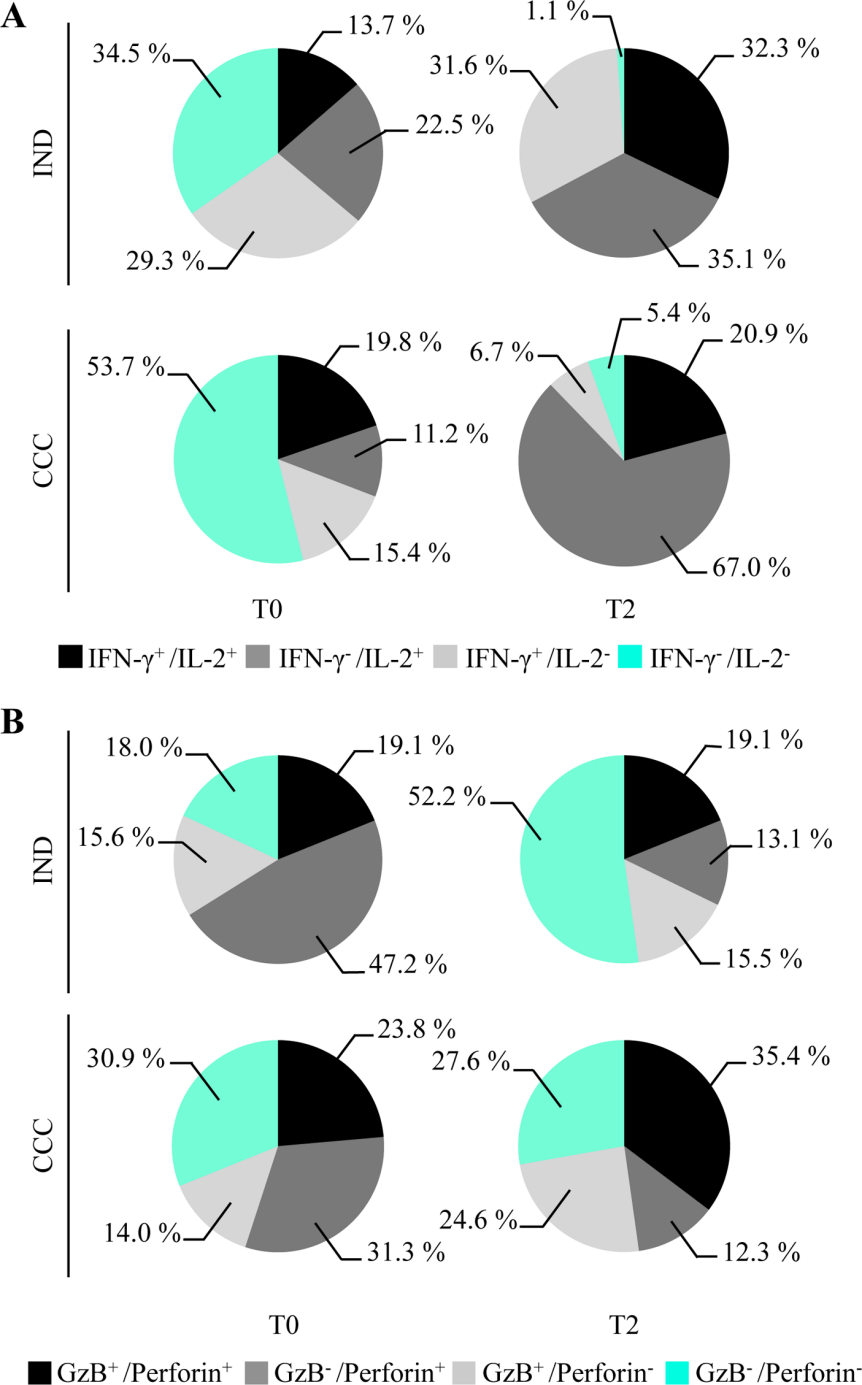
Impact of treatment in the production of cytokines or cytotoxic molecules by CD4^+^CD8^+^ T cells. The longitudinal study was carried out in IND and CCC patients **(A)** Study of the impact of the treatment on cytokine production (IFN-γ and IL-2) in response to *Tc*SA. CD4^+^CD8^+^ T cells were studied pre- (T0) and post-treatment (T2). **(B)** Impact of the treatment on the cytotoxic profile of CD4^+^CD8^+^ T cells in IND and CCC. Co-production of perforin and granzyme B against *Tc*SA before (T0) and after treatment (T2). The cChD was grouped into IND (n = 9) and CCC (n = 10).

Regarding the co-production of the cytokines IL-2 and IFN-γ (Fig 7A), there was an increase in the proportion of CD4^+^CD8^+^ T cells co-producing both cytokines after treatment only in IND (13.7% to 32.3%), although IL-2^+^IFN-γ^-^ cells were present in IND (22.5% to 35.1%) and CCC (11.2% to 67%). CCC presented a decrease in the proportion of cells producing IFN-γ, mainly due to a decrease in the proportion of CD4^+^CD8^+^IL-2^-^IFN-γ^+^ cells (15.4% to 6.7%). Furthermore, a marked decrease was observed in the proportion of cells expressing neither IL-2 nor IFN-γ after treatment in IND (34.5 to 1.1%) and CCC (53.7 to 5.4%). Analysis of the cytotoxic profile (co-production of granzyme B and perforin) revealed a reduction in the individual expression of perforin (CD4^+^CD8^+^granzyme B^-^ perforin^+^) after treatment with BNZ in IND (47.2% to 13.1%) and CCC (31.3% to 12.3%) (Fig 7B). Moreover, CCC showed a stronger cytotoxic profile after treatment for the major proportion of *T*. *cruzi* antigen-specific CD4^+^CD8^+^Granzyme B^+^Perforin^+^ T cells (23.8% to 35.4%), and an increase in CD4^+^CD8^+^Granzyme B^+^Perforin^-^ T cells (14% to 24.6%). The proportions of the subsets CD4^+^CD8^+^Granzyme B^+^Perforin^+^ and CD4^+^CD8^+^Granzyme B^+^Perforin^-^ were not altered after treatment in IND.

### Parasite detection in the peripheral blood of Chagas disease patients by PCR

Before benznidazole treatment, *T*. *cruzi* DNA was detected in 11 out of 20 IND (55%) and 9 out of 18 CCC (50%). At the end of treatment and also 24 months after treatment, all these patients had a negative PCR result.

## Discussion

A higher frequency of peripheral CD4^+^CD8^+^ T cells has been described in several pathologies, such as chronic infections, *versus* healthy donors (HD) [39–41]. An increase in the frequency of this type of cells in chronic Chagas disease patients (cChD) compared with HD has also been described [33, 42]. However, only a few studies have been performed to assess this peripheral T cell subpopulation in the context of *T*. *cruzi* infection. CD4^+^CD8^+^ T cells have been characterized as mature cells based on their expression of activation markers, their ability to respond to specific antigens and their capacity to migrate to inflamed tissues [30, 32]. Consistent with these studies, the first aim of this work was to explore the frequency of this cellular population in chronic chagasic patients at different stages of disease and their functional characterization. Thus, a higher frequency of CD4^+^CD8^+^ T cells was found in cChD compared with HD, as well as a higher frequency of this cell population in asymptomatic Chagas patients (IND) *versus* CCC. In the context of CD4^+^CD8^+^ T cells, several subpopulations have been described based on the high or low expression level of a CD8 marker. The subpopulation characterized as having low CD8 expression (CD8^low^) has been described to be derived from a group of terminally differentiated effector CD4^+^ T cells [40]. In the present study, the CD4^+^CD8^low^ T cell subpopulation constituted a higher proportion in chronic Chagas disease patients compared with HD. Moreover, the subpopulation referred to as having high CD8 expression (CD8^high^) has been described as an activated population characterized by the expression of high levels of activation markers [43]. This CD4^+^CD8^high^ T cell subpopulation made up a lower proportion in chronic Chagas disease patients compared with HD. Furthermore, this imbalance in the subpopulation ratio of CD4^+^CD8^+^ T cells relative to HD was stronger in those patients who presented cardiac-related symptoms than those who were asymptomatic. These data could indicate a deterioration or inefficacy of the immune system against *T*. *cruzi* during chronic infection, as evidenced by the proportionally larger CD4^+^CD8^low^ T subpopulation derived from terminally differentiated effector cells and proportionally smaller CD4^+^CD8^high^ T cell subpopulation, which was characterized as a subset with an activated phenotype.

In chronic diseases, the persistence of pathogen-derived antigens induces an increase in the expression of inhibitory receptors, and a gradual loss of the response capacity of antigen-specific T cells has been described. This process is known as an exhaustion process and has been observed in both CD4^+^ and CD8^+^ T cell population [44]. The exhaustion process occurs gradually, by step-wise, and includes the loss of the effector capabilities of the antigen-specific T cells together to the loss of self-renewal abilities related to the expression and co-expression of different inhibitory receptors. All this marks the degree of cell exhaustion, as a partial or severe process [44]. Interestingly, and for the first time, it is described that the population of CD4^+^CD8^+^ T cells from Chagas disease chronic patients undergoes a similar exhaustion process to that previously described in CD8^+^ [23] and CD4^+^ T cells [22, 45, 46]. Thus, a higher level of expression and co-expression of inhibitory receptors was detected in CD4^+^CD8^+^ T cells from chronic patients than in those from healthy donors. The different degrees of co-expression of inhibitory receptors observed in the evaluated population indicate that there is a mixture of functional, partially exhausted and severe exhausted CD4^+^CD8^+^ T cells. The CD4^+^CD8^+^ T cell exhaustion process observed in chronic chagasic patients could be associated with the *T*. *cruzi* antigenic persistence that occurs during the chronic phase of Chagas disease [47]. Indeed more than 50% of patients had a positive PCR result for parasite detection. The CD4^+^CD8^+^ T cell population in symptomatic patients displayed increased TIM-3 expression, as well as an increase in a very small subpopulation that co-expressed 5 exhaustion molecules. This expression pattern could be indicating a possible positive correlation between the exhaustion process and the severity of disease. We suggest that the cellular exhaustion process and, consequently, the failure in the T-cell response could lead to the loss of parasite control and evolution of the disease towards the symptomatic phase. In chronic viral infections, greater expression of inhibitory molecules by T cell is positively correlated with severe exhaustion and usually with the viral burden [44].

Elevated expression of TIM-3 by CD4^+^CD8^+^ T cells from symptomatic compared with asymptomatic Chagas disease patients has been observed. The population of CD8^+^ T cell expressing TIM-3 has been described in tumors as the most exhausted or dysfunctional population within CD8^+^PD-1^+^ T cells [48]. It has also been reported that TIM-3 plays a key role in inhibiting the expression of Th1 cytokines such as TNF-α and IFN-γ [49]. In agreement with this, the obtained results show that the CD4^+^CD8^+^ T cells from CCC have a greater expression of TIM-3 and lower percentage of TNF-α and IFN-γ production. The expression of CD160 and 2B4, outside the context of co-expression associated with the exhaustion process, was increased in CD4^+^CD8^+^ T cells from IND patients. In chronic viral infection, it has been reported that the CD160 expression improves proliferation and cytotoxic activity of CD8^+^ T cells [50]. It has been proposed that an elevated expression of CD160 and 2B4 delineated a population of cytolytic CD8^+^ T cells relevant for the control of HIV [51]. Moreover, IFN-γ secretion by NK cells was markedly reduced in CD160 (-/-) mice [52]. Consequently, it can be hypothesized that IND patients exhibit greater expression of CD160 mainly due to the relatively high proportion of CD4^+^CD8^high^ T cells (S3 Fig) which exhibit an activated phenotype.

In the absence of markers of cure for Chagas disease, it is difficult to determine whether the treatment of chronic patients is effective. Several studies have demonstrated that following treatment of chronic chagasic patients, a beneficial modification was observed in the response of CD4^+^ and CD8^+^ T cells due to the mitigation of their exhaustion processes [8, 22, 24]. Interestingly, the present results indicated that benznidazole treatment affected the CD4^+^CD8^+^ T cell population by partially reverting the exhaustion process with a reduction of the expression and co-expression of inhibitory receptors by CD4^+^CD8^+^ T cells in asymptomatic and symptomatic chronic patients. Thus, it is observed that the expression of CD160 decreased in IND and CCC and TIM-3 in CCC. Recently, a decrease of the expression of different inhibitory receptors (such as, CD160, TIM-3, CTLA-4 and PD-1) by CD8^+^ T cells has also been described in asymptomatic patients after antiparasitic treatment [24]. Herein, the individual expression of CD160 by CD4^+^CD8^+^ T cells decreased after benznidazole treatment in IND and CCC while the IL-2 production increased. In this sense, it has been described that CD160 molecule acts as inhibitory receptor independently of PD-1 and its blockage leads to the improvement of the IL-2 production by CD8^+^ T cells in chronic HIV [53]. In contrast, after treatment, the percentage of CD4^+^CD8^+^ T cells expressing CTLA-4 increased in CCC. For the latter reason, the increased numbers of cells expressing CTLA-4 was not associated with the enhancer of the cellular exhaustion process. It is assumed that this increase is related to an increase in regulatory function because this marker is fundamental for appropriate immune homeostasis [54]. These results support that benznidazole treatment reverses the exhaustion process, as previously described in CD8^+^ T cells in chronic patients [24]. Furthermore, the observed pattern of the frequency of CD4^+^CD8^+^ T cells from Chagas disease patients, expressing some inhibitory receptor is similar to that reported by the CD8^+^ T cells [23]. Otherwise, some differences in the frequency patterns of CD4^+^CD8^+^ *versus* CD8^+^ T cells expressing inhibitory receptors was observed depending on the state of disease severity. Thus, the IND had a higher percentage of CD4^+^CD8^+^ T cells expressing CD160 or 2B4 than that observed in CCC, which was not found in the CD8^+^ T cells [23]. In this context, it has been described that the pattern of co-expression of inhibitory receptors and the number of receptors simultaneously expressed by the same CD8^+^ T cells from patients with chronic viral (HIV) infection can substantially affect the severity of dysfunction [55]. Moreover, in HIV patients the CD160 was expressed by a low percentage of exhausted CD8^+^ T cells and always associated with co-expression of other inhibitor receptors [55].

It is interesting that the treatment with benznidazole increased the subpopulation of CD4^+^CD8^high^ T cells while simultaneously reduced the CD4^+^CD8^low^ T cell subpopulation. CD4^+^CD8^high^ T cells have been described to be a more activated population of CD4^+^CD8^+^ T cells when HLA-DR and CD38 activated markers are measured in the context of Chagas disease [33]. Some studies have asseverated that CD4^+^CD8^low^ T cells derive from the terminal senescence of the CD4^+^ T cell population [40], as mentioned previously. Our results showed that benznidazole treatment reduced the CD4^+^CD8^low^ T cell subpopulation and increased the number of CD4^+^CD8^high^ T cells, reaching a similar ratio than that observed in HD. Thus, the treatment effect in terms of the ratio of CD4^+^CD8^+^ T cell subpopulations could be important for achieving a better response of this subset of cells for the control of *T*. *cruzi* infection.

In the present manuscript, it is shown that CD4^+^CD8^+^ T cells exhibited an antigen-specific cytotoxic ability against the parasite, co-expressing granzyme B and perforin. The expression of both molecules is critical for effective cytotoxic action against *T*. *cruzi* in tissues [56]. Furthermore, the CD4^+^CD8^+^ T cells also presented a Th1 antigen-specific response since they produced cytokines such IFN-γ and IL-2. The Th1 profile has been shown to function in favor of the control of *T*. *cruzi* infection and is related to a mild form of Chagas disease [23, 57]. It has been described that a greater proportion of CD4^+^CD8^+^ T cells from healthy donors has an enhanced capacity to produce cytokines (such as IFN-γ, TNF-α, IL-2) and cytotoxic molecules (like perforin and granzyme B) compared to CD4^+^ and/or CD8^+^ T lymphocytes [30]. In the context of chronic infections, such as HIV, an increased production of IFN-γ by antigen-specific CD4^+^CD8^+^ T cells has also been reported in comparison with other T cell populations [29]. In chronic Chagas disease, an exceptionally high percentage of CD4^+^CD8^+^ T cells specific of K1 peptide (a *T*. *cruzi* immunodominant cytotoxic epitope [58, 59] has been previously described [33] compared to the K1-specific CD8^+^ T cell population [23]. The results shown here indicate that in IND the frequency of antigen-specific CD4^+^CD8^+^ T cells that produce IFN-γ or IL-2 is higher than that of the antigen-specific CD8^+^ T cells [24]. Likewise, in these IND there is a higher percentage of antigen-specific CD4^+^CD8^+^ T cells exhibiting a multifunctional profile than that reported for the CD8^+^ T cells [23]. The biological significance of the slightly higher frequency of CD4^+^CD8^+^ T cells expressing IFN-γ or TNF-α detected in IND *versus* CCC and the higher frequency of CD4^+^CD8^+^ T cells expressing granzyme B in CCC *versus* IND, should be considered in the context of multifunctionality of these cells. In fact, we observed a slightly greater multifunctional capacity of CD4^+^CD8^+^ T cells based on the higher proportion of cells that co-produce 3 molecules and a higher frequency of CD4^+^CD8^high^ cells in IND patients than in CCC. Furthermore, in qualitative terms, these cells from IND but not those from CCC mainly exhibit a Th1-type cytotoxic profile expressing IFN-γ, perforin and granzyme B. Consequently, we suggest that the antigen-specific CD4^+^CD8^+^ T cells could play an important role in the control of *T*. *cruzi* infection, as previously described for other T cell populations [27, 60–62].

Benznidazole treatment improved the cellular capacity to respond to specific antigens in CD4^+^CD8^+^ T cells and increased the frequency of *T*. *cruzi* antigen-specific CD4^+^CD8^+^ T cells producing IL-2 in IND and CCC, and TNF-α in IND. Furthermore, the treatment enhanced the multifunctional capacity of antigen-specific CD4^+^CD8^+^ T cells. These changes occurred in parallel to the decrease in inhibitory receptors co-expression, which could be related to reversion of the T-cell exhaustion process and, thus, improvement of the cellular functionality against the parasite-derived antigens. Interestingly, the recovery of cytokine production in CD4^+^CD8^+^ T cells occurred in a stepwise manner in an order opposite to that observed during the T-cell exhaustion process, in which cytokine production was lost in the following order: IL-2>TNF-α>IFN-γ [63, 64]. Thus, our results showed that after treatment, IL-2 production was recovered in both IND and CCC, TNF-α production was recovered in IND and started to recover in CCC, and IFN-γ production started to recover in IND. Likewise, antiparasitic treatment reduced the proportion of CD4^+^CD8^+^Perforin^+^Granzyme B^-^ T cells but maintained a population of CD4^+^CD8^+^ T cells capable of co-producing perforin and granzyme B. This observation could be important because of the results of previous studies have shown that the cytolysis process mediated only by perforin can be less efficient [65] and a key component of the potential tissue damage [66, 67]. A cytotoxic profile that co-expresses both molecules (perforin and granzyme B) combined with cytokine production has been described for CD8^+^ T cells in patients with a mild form of Chagas disease [23]. This multifunctional profile is enhanced after benznidazole treatment in CD8^+^ T cells [24].

The analysis of the parasite load in the peripheral blood showed presence of the parasite in approximately 50% of the patients independently of the phase of the disease. At the end of the treatment and 24 months after treatment all patients presented a negative PCR result. According to the PCR data obtained in this study, are those previously reported that support the effect of the benznidazole on the reduction of the parasite load [12]. In spite of that, and the fact that a PCR positive result is consistent with treatment failure, a negative PCR cannot be associated to treatment success [47]. Consequently, neither the obtained data regarding to the parasite load detected by PCR nor the existence of clinical changes associated to progression (or not) of the sickness in these patients allowed to evaluate the efficacy of benznidazole treatment.

In summary, these findings suggest that CD4^+^CD8^+^ T cells could be an important component of the immune response against *T*. *cruzi* infection during the chronic phase of Chagas disease. These cells are able to produce a multifunctional response against the parasite and undergo an exhaustion process associated to a long period of coexistence of the parasite with its host. These findings reflected in this work, shed light on the understanding of a population that is poorly described at a functional level in the context of Chagas disease. Benznidazole treatment reduces the CD4^+^CD8^low^ T cell and increases the CD4^+^CD8^high^ T cell subpopulations. Furthermore, this treatment improves the quality of antigen-specific CD4^+^CD8^+^ T cell responses and partially reverses the exhaustion process of these cells. Importantly, these results add to our knowledge concerning the immune response against *T*. *cruzi* infection, potentially providing a tool to analyze the efficacy of benznidazole treatment in patients with chronic Chagas disease.

## Supporting information

S1 Fig Representative plots of the gating strategy used. **(A)** Gating strategy used to analyze CD4^+^CD8^+^ T cells and subpopulations of those cells: CD4^+^CD8^high^ and CD4^+^CD8^low^. **(B)** Gating strategy used to analyze inhibitory receptor expression of CD4^+^CD8^+^ T cells. **(C)** Gates to analyze the functional activity of CD4^+^CD8^+^ T cells. PBMC samples were acquired by flow cytometry and analyzed using FlowJo 9.3.2 software.
(TIF)

S2 Fig Expression levels of 2B4, CD160, CTLA-4, PD-1 and TIM-3 in CD4^+^CD8^+^ T cells measured by the mean fluorescence intensity (MFI) in cChD (IND and CCC) and HD. **(A)** MFI of 2B4, CD160, CTLA-4, PD-1 and TIM-3 in CD4^+^CD8^+^ T cells from cChD and HD. **(B)** Expression levels of the inhibitory receptors in CD4^+^CD8^+^ T cells from IND, CCC and HD. Statistical analyses were carried out using the Mann-Whitney U test. Statistically significant differences are indicated by (*) ρ<0.05, (**) ρ<0.01, (***) ρ<0.001 and (****) ρ<0.0001. Study population grouped by cChD (IND (n = 19) and CCC (n = 16)) and HD (n = 12).
(TIF)

S3 Fig Percentage of CD4^+^CD8^high^ and CD4^+^CD8^low^ T cells expressing CD160 in IND and CCC. Statistical analyses were carried out using the Mann-Whitney U test. Statistically significant differences are indicated by (*) ρ<0.05 and (****) ρ<0.0001. Study population grouped by cChD (IND (n = 19) and CCC (n = 16)) and HD (n = 12). The cChD was grouped into IND (n = 18) and CCC (n = 16).
(TIF)
