# Supplementary figures and images for: Impact of benznidazole treatment on the functional response of Trypanosoma cruzi antigen-specific CD4+CD8+ T cells in chronic Chagas disease patients
Source: PLoS Negl Trop Dis. 2018 May 11;12(5):e0006480. doi: 10.1371/journal.pntd.0006480 (PMC5965897; doi:10.1371/journal.pntd.0006480)

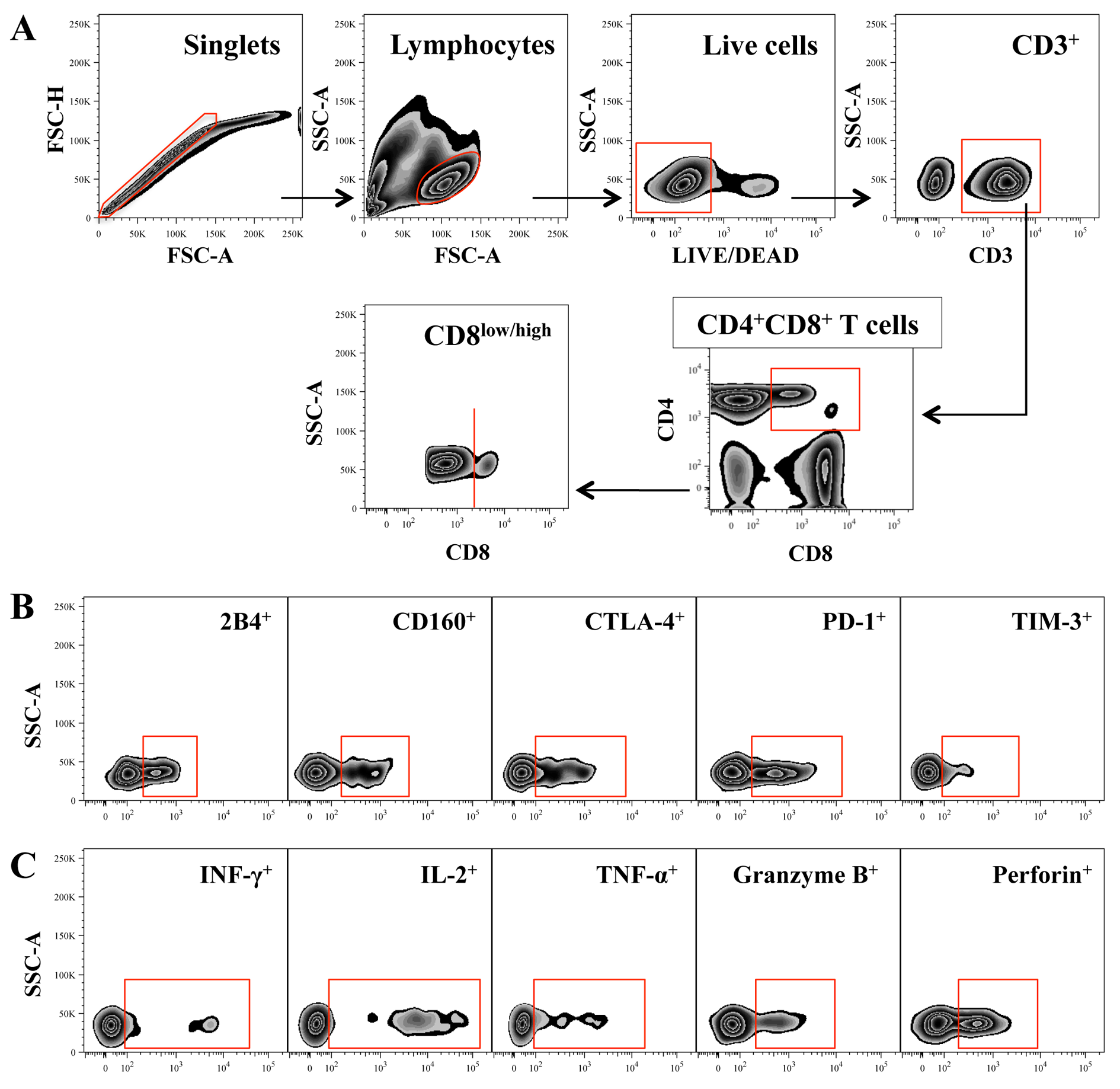

Supplement: S1 Fig — (A) Gating strategy used to analyze CD4+CD8+ T cells and subpopulations of those cells: CD4+CD8high and CD4+CD8low. (B) Gating strategy used to analyze inhibitory receptor expression of CD4+CD8+ T cells. (C) Gates to analyze the functional activity of CD4+CD8+ T cells. PBMC samples were acquired by flow cytometry and analyzed using FlowJo 9.3.2 software. (TIF) [file pntd.0006480.s001.tif]

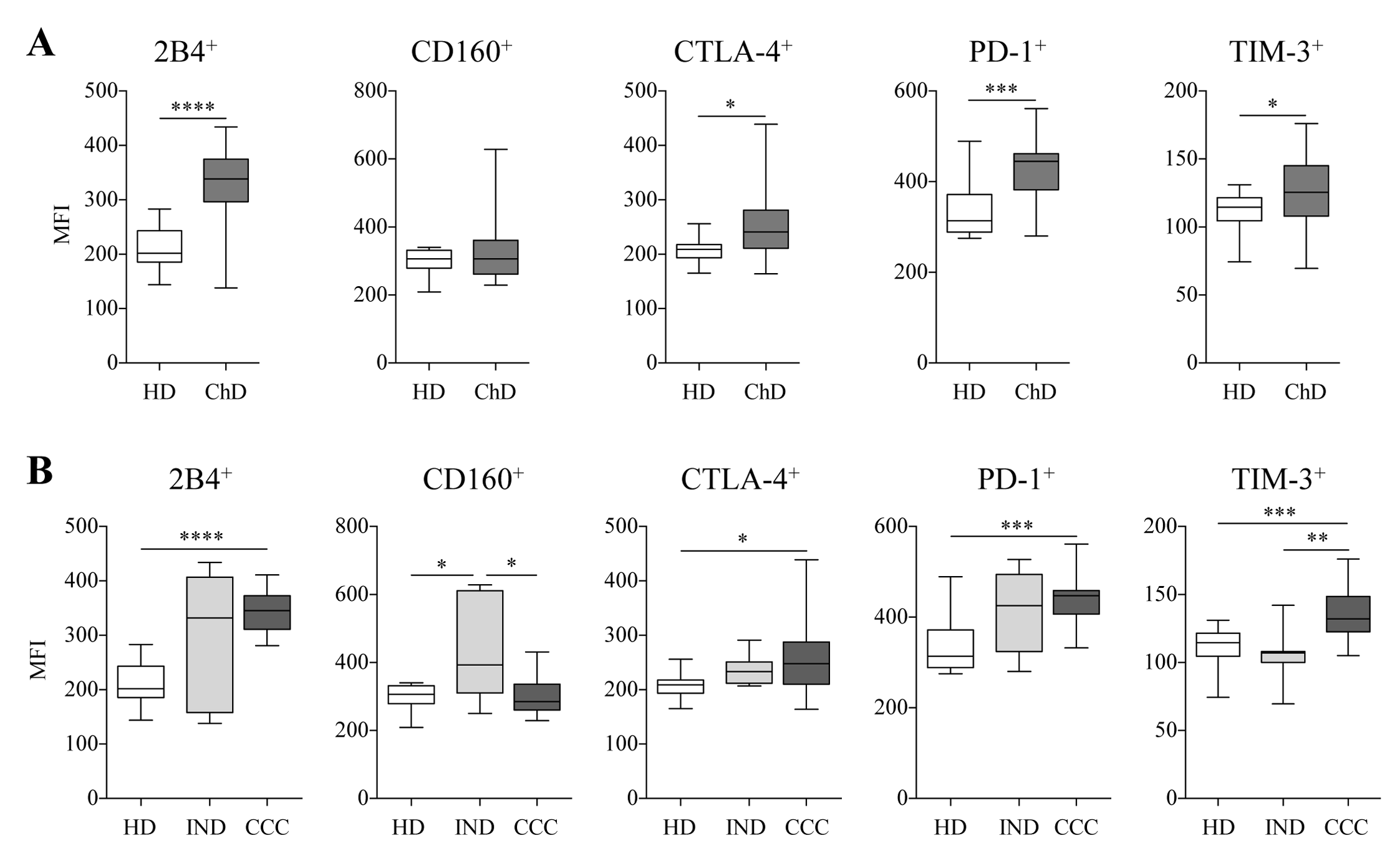

Supplement: S2 Fig — (A) MFI of 2B4, CD160, CTLA-4, PD-1 and TIM-3 in CD4+CD8+ T cells from cChD and HD. (B) Expression levels of the inhibitory receptors in CD4+CD8+ T cells from IND, CCC and HD. Statistical analyses were carried out using the Mann-Whitney U test. Statistically significant differences are indicated by (*) ρ<0.05, (**) ρ<0.01, (***) ρ<0.001 and (****) ρ<0.0001. Study population grouped by cChD (IND (n = 19) and CCC (n = 16)) and HD (n = 12). (TIF) [file pntd.0006480.s002.tif]

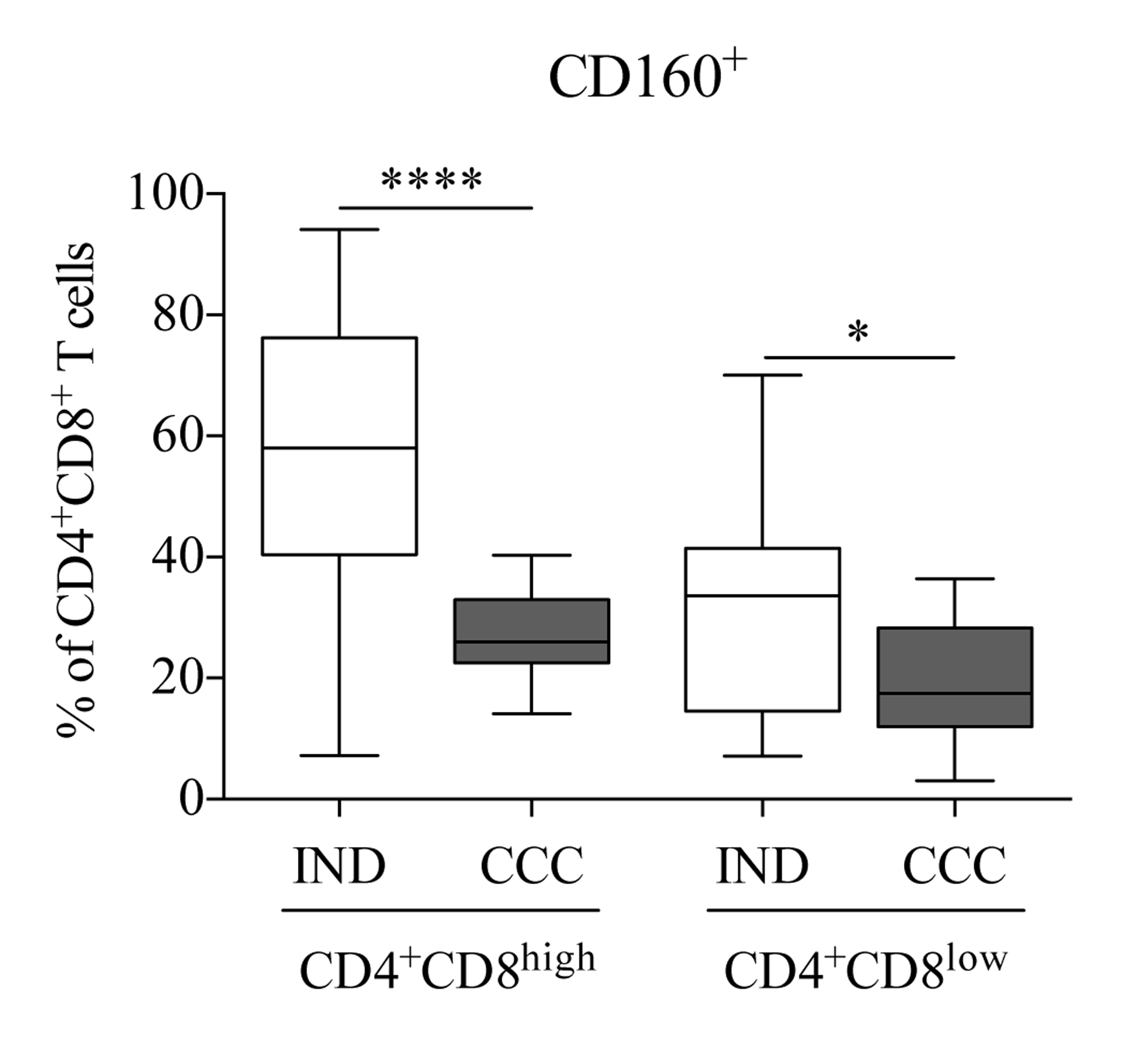

Supplement: S3 Fig — Statistical analyses were carried out using the Mann-Whitney U test. Statistically significant differences are indicated by (*) ρ<0.05 and (****) ρ<0.0001. Study population grouped by cChD (IND (n = 19) and CCC (n = 16)) and HD (n = 12). The cChD was grouped into IND (n = 18) and CCC (n = 16). (TIF) [file pntd.0006480.s003.tif]
